# Ketosis After Intake of Coconut Oil and Caprylic Acid—With and Without Glucose: A Cross-Over Study in Healthy Older Adults

**DOI:** 10.3389/fnut.2020.00040

**Published:** 2020-04-15

**Authors:** Jakob Norgren, Shireen Sindi, Anna Sandebring-Matton, Ingemar Kåreholt, Makrina Daniilidou, Ulrika Akenine, Karin Nordin, Staffan Rosenborg, Tiia Ngandu, Miia Kivipelto

**Affiliations:** ^1^Division of Clinical Geriatrics, Department of Neurobiology, Care Sciences and Society (NVS), Center for Alzheimer Research, Karolinska Institutet, Stockholm, Sweden; ^2^Neuroepidemiology and Ageing Research Unit, School of Public Health, Imperial College London, London, United Kingdom; ^3^Division of Neuro Geriatrics, Department of Neurobiology, Care Sciences and Society (NVS), Center for Alzheimer Research, Karolinska Institutet, Stockholm, Sweden; ^4^Department of Neurobiology, Care Sciences and Society (NVS), Aging Research Center, Karolinska Institutet and Stockholm University, Stockholm, Sweden; ^5^School of Health and Welfare, Institute of Gerontology, Aging Research Network-Jönköping (ARN-J), School of Health and Welfare, Jönköping University, Jönköping, Sweden; ^6^Theme Aging, Karolinska University Hospital, Stockholm, Sweden; ^7^Clinical Pharmacology, Karolinska University Hospital, Stockholm, Sweden; ^8^Public Health Promotion Unit, Department of Public Health Solutions, Finnish Institute for Health and Welfare, Helsinki, Finland; ^9^Department of Neurology, Institute of Clinical Medicine and Institute of Public Health and Clinical Nutrition, University of Eastern Finland, Kuopio, Finland; ^10^Research and Development Unit, Stockholms Sjukhem, Stockholm, Sweden

**Keywords:** ketosis, β-hydroxybutyrate, aged, glucose, medium-chain fatty acids, coconut oil, ketogenic diet, fasting

## Abstract

**Introduction:** Medium-chain-triglycerides (MCT), formed by fatty acids with a length of 6–12 carbon atoms (C6–C12), constitute about two thirds of coconut oil (Coc). MCT have specific metabolic properties which has led them to be described as ketogenic even in the absence of carbohydrate restriction. This effect has mainly been demonstrated for caprylic acid (C8), which constitutes about 6–8% of coconut oil. Our aim was to quantify ketosis and blood glucose after intake of Coc and C8, with and without glucose intake. Sunflower oil (Suf) was used as control, expected to not break fasting ketosis, nor induce supply-driven ketosis.

**Method:** In a 6-arm cross-over design, 15 healthy volunteers—age 65–73, 53% women—were tested once a week. After a 12-h fast, ketones were measured during 4 h after intake of coffee with cream, in combination with each of the intervention arms in a randomized order: 1. Suf (30 g); 2. C8 (20 g) + Suf (10 g); 3. C8 (20 g) + Suf (10 g) + Glucose (50 g); 4. Coc (30 g); 5. Coc (30 g) + Glucose (50 g); 6. C8 (20 g) + Coc (30 g). The primary outcome was absolute blood levels of the ketone β-hydroxybutyrate, area under the curve (AUC). ANOVA for repeated measures was performed to compare arms.

**Results:** β-hydroxybutyrate, AUC/time (mean ± SD), for arms were 1: 0.18 ± 0.11; 2: 0.45 ± 0.19; 3: 0.28 ± 0.12; 4: 0.22 ± 0.12; 5: 0.08 ± 0.04; 6: 0.45 ± 0.20 (mmol/L). Differences were significant (all *p* ≤ 0.02), except for arm 2 vs. 6, and 4 vs. 1 & 3. Blood glucose was stable in arm 1, 2, 4, & 6, at levels slightly below baseline (*p* ≤ 0.05) at all timepoints hours 1–4 after intake.

**Conclusions:** C8 had a higher ketogenic effect than the other components. Coc was not significantly different from Suf, or C8 with glucose. In addition, we report that a 16-h non-carbohydrate window contributed to a mild ketosis, while blood glucose remained stable. Our results suggest that time-restricted feeding regarding carbohydrates may optimize ketosis from intake of MCT.

**Clinical Trial Registration:** The study was registered as a clinical trial on ClinicalTrials.gov, NCT03904433.

## Introduction

The interest for ketosis as a potentially beneficial metabolic state for health started in the epilepsy field in the 1920's (1), and in more recent decades the interest has expanded to a broad range of neurological conditions (2), including Alzheimer's disease (3), and also to weight loss (4), type 2 diabetes (5), and high performance in sports (6). Fasting or carbohydrate-restricted diets are typically used to induce ketosis, which is a condition where the liver uses fatty acids to produce the ketone bodies (“ketones”) β-hydroxybutyrate (BHB) and acetoacetate (AcAc). Ketones are transported through the blood and provide energy for various tissues, which is essential for the brain, due to its limited ability to extract energy directly from fatty acids (7). Ketones also have a function as substrate for lipid synthesis (8), and in recent years BHB has been identified as an epigenetic signal molecule related to brain health (9).

Medium-chain fatty acids (MCFA)—which form together as medium-chain triglycerides (MCT)—have been described as providing a shortcut to ketosis, by their specific metabolic properties compared to long-chain fatty acids (LCFA), which dominate human diets. However, as pointed out by Dayrit (10), the concept MCT is used inconsistently and sometimes without definition in the literature. Sometimes it refers to a triglyceride formed by fatty acids with a carbon chain of 6–12 atoms, which is the chemical definition, including caproic (C6), caprylic (C8), capric (C10), and lauric (C12) acid. However, often MCT refers to just C8 and C10, which is the content of many commercialized “MCT-oils,” used as dietary supplements since the 1960's. This has led to an ambiguity on whether C12 share the ketogenic properties of the shorter MCFA. As coconut oil is constituted by almost 50% C12 and just ≈6–8% each of C8 and C10, the ketogenic effect of coconut oil has also been unclear. A recent intervention study investigated the acute ketogenic response to coconut oil and different MCFA-combinations, including ≥91% concentrations of C8, C10, and C12. Comparisons of the arms have been reported in two articles (11, 12), concluding that C8 increased blood ketones during a few hours, while the ketogenic response to the other MCFA and coconut oil was weak. In the current article the concepts MCFA/MCT will refer to C6–C12, but while C6 only constitutes <1% of coconut oil, and is rarely used in supplements, it will not be further discussed.

Fasting and caloric restriction in different forms have been associated with a broad range of health and longevity pathways. Several of the same pathways have also been associated with ketogenic diets, and in some of them BHB has been identified as a mediator (9, 13, 14). Time restricted feeding is a form of intermittent fasting, where total energy intake is not necessarily reduced, but intake is concentrated to e.g., 8 h per day, leaving a 16-h daily fasting period. The combination of MCT intake and a time restricted eating pattern could potentially enhance ketosis, as carbohydrates inhibit ketosis through an increase in insulin secretion and a decrease in glucagon (15). In ketogenic diet protocols, the daily carbohydrate limit to avoid breaking ketosis is often set in the range 20–50 g (16). The ketogenic response to fasting is much quicker in children compared to adults (17), but whether the ketogenic response is influenced by aging is not well studied in humans. Here we study a sample of older adults, with the purpose of optimizing future ketogenic interventions in the field of cognitive health.

The first aim of this study was to compare the short-term ketogenic effect of coconut oil vs. C8 for 4 h, and to investigate how this response is affected by previous intake of 50 g of glucose. In contrast to previous studies, which have mainly investigated change in ketones vs. baseline levels, our main interest is the absolute level of ketosis. We expect this outcome to give clarity on the interaction between ketogenic and anti-ketogenic processes. The second aim was to investigate the response in blood glucose to these interventions. The third aim was to assess satiety and tolerance, to evaluate the feasibility of investigating a similar administration of the fatty acids in long-term interventions. Additionally, we will analyze inter-individual differences in the BHB responses to C8.

## Materials and Methods

### Study Sample

Fifteen healthy volunteers, 53% women, were recruited by advertising in a daily newspaper. Inclusion criteria were age 65–75 years, written informed consent during a screening visit, and daily coffee consumption, as coffee was used as a vehicle. Exclusion criteria were weight <50 kg, current smoking, diagnosed diabetes (type 1 or 2), history of heart disease, history of disease related to internal organs or metabolism, experience of “sensitive gut” or known intolerance to coconut oil or sunflower oil, medication expected to affect glucose- or lipid-metabolism, fasting during the study or one month before, high intensity physical activity >3 times/week, dementia, severe psychiatric conditions, Hb <125 g/L, and participation in a lifestyle intervention during the last 6 months. Baseline characteristics of the participants are described in Table 1. Body mass index (BMI) and age were assessed at the screening visit. The other values are from the first study day, after a 12 h overnight fast. Participants were informed that fatty acids from coconut oil and sunflower would be used in the study, but were blinded from further details. The sample size was calculated based on effect sizes reported in previous studies (12), and was supposed to be sufficient to detect differences of clinical significance.

**Table 1:** Characteristics of the participants.

**Age (years)**	**69.2 ± 2.4**
BMI (kg/m^2^)	23.9 ± 4.0
Glucose (mmol/L)	5.2 ± 0.6
Insulin (mIE/L)	5.5 ± 3.3
Glucagon (pmol/L)	40.3 ± 4.4
BHBv (mmol/L)	0.15 ± 0.14
BHBp (mmol/L)	0.12 ± 0.08
Acetoacetate (mmol/L)	0.18 ± 0.03
Total ketones (mmol/L)	0.30 ± 0.10

*Mean values and standard deviations. BHBp, β-hydroxybutyrate in plasma; BHBv, β-hydroxybutyrate in venous whole blood*.

### Study Design

In a cross-over design, participants were exposed to six different intervention arms, which are described and labeled with abbreviations in Table 2. Participants were randomized to receive the intervention arms weekly in one of the following sequences: 421653, 216435, 164253, 642135. The test oils were mixed with 2.5 dl coffee—containing ~170 mg caffeine—and 15 g full-fat cream, containing 0.4 g carbohydrates, 0.3 g protein and 6 g fat (≤0.6 g MCT). Caffeine has been reported to have a mild ketogenic effect, mainly 3–4 h after intake when consumed at levels comparable to those in our study (18). We expect the relative ketogenic contribution from caffeine in our study to be small, and equally distributed between arms. Cream was added for the purpose of masking any nuances of the test oils, and it may also give some contribution to satiety. At the given dose we expect cream to have a negligible effect on ketosis. Sunflower oil is not assumed to be an active substance of arm 2 & 3 (19), but is added to balance caloric content, and potential competition in uptake of MCFA by LCFA. Therefore, the labeling will be just C8. Arm 1, 2, 4, & 6—referred to as the non-glucose arms—include a 16-h non-carbohydrate window, which can be described as time-restricted feeding regarding carbohydrates. Although this technically also could be labeled a non-protein window, our labeling is based on the fact that carbohydrates are the main inhibitors of ketosis (16, 20). Arm 1, 2, & 4 will be referred to as low-caloric, and 3, 5, & 6 as high-caloric.

**Table 2:** Description of the intervention arms, and introduction of abbreviations.

	**Intervention Arms (intake in combination with 2.5 dl coffee & 15 g cream)**		
Suf	1: Sunflower oil (30 g)	low-caloric	0% MCT
C8	2: C8 (20 g) + Sunflower oil (10 g)	low-caloric	67% MCT (C8)
C8+Glu	3: Glucose (50 g)— [15 min] = => C8 (20 g) + Sunflower oil (10 g)	high-caloric	=Arm 2 + glucose
Coc	4: Coconut oil (30 g)	low-caloric	≈62% MCT (mainly C12)
Coc+Glu	5: Glucose (50 g)— [15 min] = => Coconut oil (30 g)	high-caloric	=Arm 4 + glucose
**C8+Coc**	6: Coconut oil (30 g) + C8 (20 g)	high-caloric	MCT-content of arm 2 + 4

*Low-caloric ≈ 300 kcal; high-caloric ≈ 500 kcal*.

*Arm 1: Black was selected to represent the “control” arm*.

*Arm 2 and 4: Red and sand were arbitrarily chosen colors for C8 and Coc*.

*Arm 3 and 5: These arms correspond to 2 and 4, respectively (with glucose added). Colors are therefore the same as 2 and 4, but with lower opacity (in Figures 2, 5 they are striped, instead of changing opacity)*.

*Arm 6: Blue was chosen arbitrarily as a contrasting color*.

### Test Oils

The test ingredients were bought in local food or health stores, and were from arbitrarily chosen brands: Sunflower oil, constituted by 100% LCFA. C8 (100%) from highly refined coconut oil, which means 100% MCFA. Deodorized coconut oil with fatty acid composition in the following ranges, according to the manufacturer: C8 4.6–10%, C10 5.5–8.0%, C12 45.1–50.3%. By assuming the midpoint of the ranges, we calculated MCFA content to ≈62%, and thus LCFA content to ≈38%.

### Sampling Procedure and Laboratory Analyses

Recruitment and data collection took place between August and October 2018. The study was conducted at the Clinical Pharmacology Trial Unit (CPTU) at the Karolinska University Hospital, Huddinge, Sweden. One participant, exposed to the Coc arm, dropped out during the first study week due to reporting severe diarrhea which occurred several hours after leaving the study center. That participant was replaced by another from the waiting list, who was exposed to all intervention arms. Participants were instructed to keep their usual habits regarding diet and exercise, and to especially avoid deviation from habits on days preceding a study day. Instructions were given to consume nothing but water after 8 P.M. the day before testing, to not consume alcohol the day before testing, and to avoid physical activity exceeding 20 min stroll in the morning of a study day.

On each study day participants arrived at 7.30 A.M. They were at rest during the study session, and water was allowed *ad libitum*. A venous catheter was applied for repeated collection of blood. Baseline assessments were performed 20–40 min after arrival, and within the following 0–5 min participants received the drink in a covered cup, at a time point defined as T0. They were instructed to ingest the drink during 5–7 min. In arm 3 & 5, participants were served 50 g glucose dissolved in a glass of water, 15 min before the blood test preceding T0. The decision to place the glucose intake prior to T0 (without a baseline assessment) was based on considerations of tolerance and logistics, and it should have negligible effect on the interpretation of our primary outcome, which is area under the curve (AUC) of absolute ketone levels. However, it limits dynamic comparisons between glucose and non-glucose arms.

Details of the sampling and analyses of ketones has been described previously (21), and a summary of the procedure is given here. Our main outcome, BHB assessed in venous whole blood (BHBv), was measured with a point-of-care meter (Statstrip Xpress®) at time points T0, 15, 30, 45, 60, 75, 90, 120, 150, 180, 210, and 240 (minutes). Whole blood was drawn by a syringe through the venous catheter, and then applied to the test strip within ~10 s. BHBv was validated against a laboratory assay on total ketones, calculated from plasma BHB (BHBp) + AcAc, at T0, 30, 60, & 120, which showed that the correlation was high (Pearson's r = 0.91, *p* < 0.0001; *n* = 360) for BHBv vs. BHBp, as well as for BHBv vs. total ketones). Also, the agreement between BHBv and BHBp was satisfactory (Lin's concordance coefficient of absolute agreement = 0.91) (21). A divergence in the ratio between BHB and AcAc has been reported after intake of C8 vs. coconut oil (11, 12), which could potentially induce bias when only BHB is measured. We therefore analyzed if a divergence could be observed in our data. Glucose was assessed at T0, 60, 120, 180, and 240. Insulin and glucagon were assessed at T0 and 240, and additionally at T0, 30, 60, and 90 in the glucose-arms (3 & 5).

Glucose, insulin, glucagon, BHBp, and AcAc were analyzed according to routine procedures at two different university hospital laboratories. Glucose was analyzed from venous blood by enzymatic photometry and potentiometry. Insulin was analyzed from serum by immunochemistry, electrochemilumiscent. Glucagon was analyzed from plasma by immunochemistry (RIA). BHBp levels were measured using the Randox D-3 Hydroxybutyrate (Ranbut) reagent kit (Randox, Crumlin, UK). Quantification of BHBp was performed with an automated clinical chemistry analyzer (ABX Pentra C400, Horiba, Montpellier, France) using a spectrophotometric endpoint assay at 340 nm and 37°C. AcAc was measured by mixing 8 μl of plasma diluted in 5 μl H_2_O with 153 μl working reagent containing 2.3 U 3-Hydroxybutyrate dehydrogenase (Sigma-Aldrich, MO, USA), 96.5 mmol/L calcium phosphate buffer, pH 7.0 (Merck, NJ, USA) and 0.18 mmol/L NADH (Sigma-Aldrich, MO, USA). The conditions and analyzers used for BHB were also used for AcAc samples. In the calculations, we used raw data for values below the ranges that had been validated at the lab—AcAc <0.20 mmol/L and BHBp <0.10 mmol/L. Storage and transportation followed the laboratory guidelines, with the exception of 9% of the AcAc samples, which were analyzed 1–2 days after the proposed 5 days at −20°C limit. However, by storing at −80° most of the time—which is expected to extend stability (22)—we assume this minor delay to be negligible. In contrast to BHBp, which was measured with two decimals, BHBv was measured with one decimal. BHBv was selected as the main outcome due to its much lower cost compared to BHBp, which enabled more frequent monitoring of ketosis.

A questionnaire, designed for this study, on satiety and tolerance was given immediately after T240 on each study day. Tolerance was assessed by this question: “Did you experience any inconvenience (e.g., nausea, upset stomach), which you attribute to the beverage you were served today?” (a. No / b. Yes, minor inconvenience. / c. Yes, moderate inconvenience. /d. Yes, major inconvenience). If they selected answers b, c, or d, they were asked to give a short description. Participants were informed that they could contact the research assistant and report inconveniences occurring after leaving the study center. Satiety was assessed by one question with five alternative answers, described in **Table 4**. The scales of the questionnaires were considered ordinal.

Our study was approved by The Regional Ethical Review Board in Stockholm, and complies with the Declaration of Helsinki. The study was registered as a clinical trial on ClinicalTrials.gov, NCT03904433.

### Statistical Analyses

The software used for all analyses was STATA 15. Calculations of AUC were performed according to the trapezoidal method. Arms were compared with ANOVA for repeated measures. When comparing specific time points we used a mixed model with *arm* × *minute* as categorical variables, controlling for subject. Correlations were determined by Pearson's *r*. Results are reported in tables and graphs with a significance level of *p* < 0.05, without correction for multiple comparisons. Bonferroni corrected *p*-values are additionally reported in the results text. Log transformation of AUC for the ketone measures increased normality, but the effect on the calculations was very small, so we kept the untransformed variables. After normality check of the other variables we decided to use log transformed values for the BHBp/AcAc-ratio, and the insulin/glucagon-ratio. Test of homogeneity of the variance for our primary outcome indicated no difference between arms 1, 2, 3, 4, & 6. The variance was lower in arm 5, possibly due to a floor effect. For glucose, there was a difference in variance between the glucose and non-glucose arms, but not between arms within those categories. We only report comparisons between the non-glucose arms.

## Results

Data was collected without missing values, with the exception of a questionnaire on tolerance and satiety missing from one participant in arm 6, and a value of AcAc in arm 5, which was replaced by the average of the values before and after (0.15 & 0.18). Only 7 out of 90 values of BHBv at T0, divided among 5 participants, was higher than 0.3 mmol/L. Five of those values were 0.4 and two were 0.6. At T0 there were no significant differences between the non-glucose arms in any of the ketone measures, glucose or the insulin/glucagon-ratio (data not shown).

**Figure 1:**
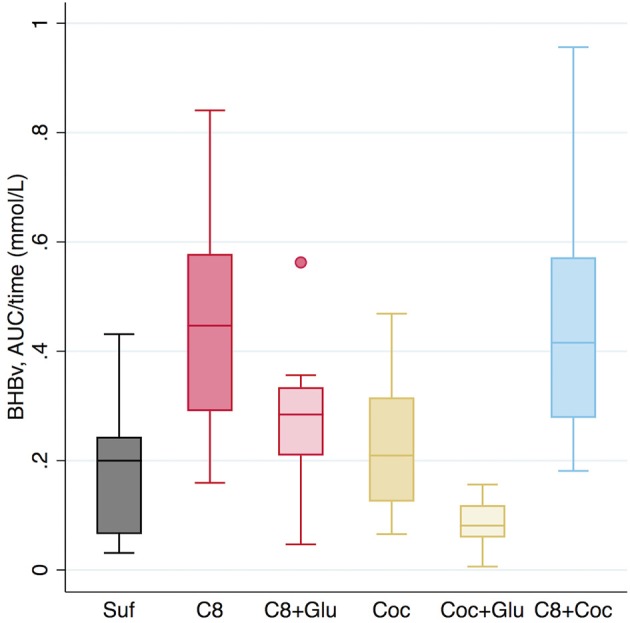
BHBv concentrations in blood, AUC/time, (mmol/L) during T0–T240 (minutes). Values are absolute and not adjusted for baseline levels. Suf, Sunflower oil (30 g); C8, Caprylic acid (20 g) + Sunflower oil (10 g); C8+Glu, Caprylic acid (20 g) + Sunflower oil (10 g) + Glucose (50 g); Coc, Coconut oil (30 g); Coc+Glu, Coconut oil (30 g) + Glucose (50 g); C8+Coc, Caprylic acid (20 g) + Coconut oil (30 g). BHBv, β-hydroxybutyrate in venous whole blood.

**Figure 2:**
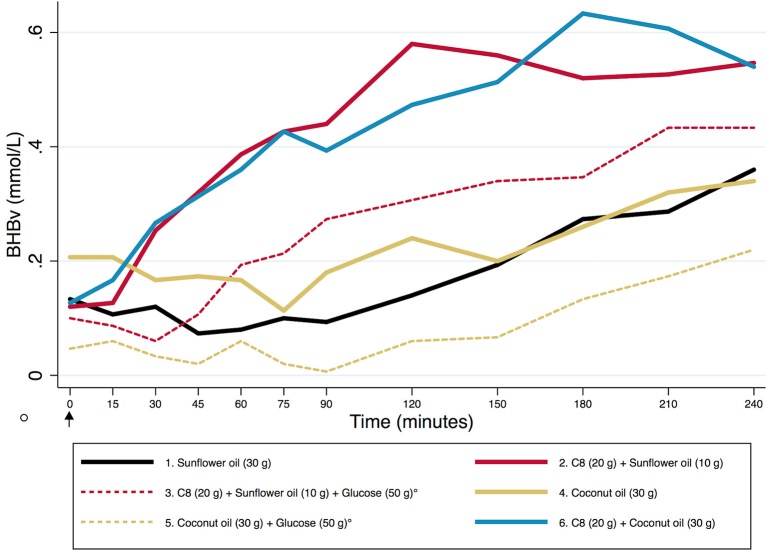
The dynamics of BHBv during T0–T240 (minutes). A descriptive graph based on absolute mean values. The arrow at T0 indicates the start of ingestion of coffee with fatty acids. The circle indicates intake of glucose (arms 3 & 5 only, striped lines), 15 min before the blood test performed immediately before T0. BHBv, β-hydroxybutyrate in venous whole blood.

**Figure 3:**
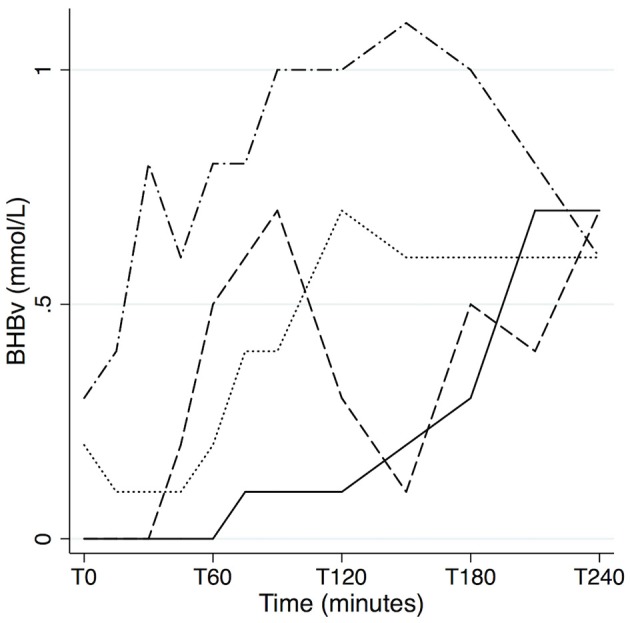
Individual differences in the ketogenic response to C8. The paths of four subjects in the C8 arm (20 g Caprylic acid + 10 g Sunflower oil), selected to illustrate the big individual differences in BHBv response, and the potentially transient nature of ketosis. BHBv, β-hydroxybutyrate in venous whole blood.

Our primary outcome, which was absolute levels of AUC (T0–T240) for BHBv, is reported in Figure 1. Values can be interpreted as mean concentrations during the 4 h, as AUC is divided by time in the graph. Means and standard deviations of BHBv, AUC/time, for the different arms were as follows: Suf: 0.18 ± 0.11; C8: 0.45 ± 0.19; C8+Glu: 0.28 ± 0.12; Coc: 0.22 ± 0.12; Coc+Glu: 0.08 ± 0.04; C8+Coc: 0.45 ± 0.20 (mmol/L). A description of the effect sizes, using Coc+Glu as a reference (100%), gives the following results: Suf: 216%, C8: 544%, C8+Glu: 336%, Coc: 273%, C8+Coc: 547%. Differences between arms are presented with 95% CI, without correction for multiple comparisons, in Table 3. All significant results remained significant (*p* ≤ 0.003) after Bonferroni correction was performed, with the exception of Suf vs. C8+Glu and Suf vs. Coc+Glu, for which the significance level was *p* < 0.10. A descriptive graph of the dynamics of BHBv is available in Figure 2. A mixed model with *arm* × *minute* as categorical variables, controlling for subject, revealed that C8 was not different from C8+Coc at any time point (all *p* > 0.15). Big individual differences in the ketogenic response to C8 were observed, which is exemplified in Figure 3 by four individuals selected to illustrate this variation.

**Table 3:** Differences (row-column) in BHBv (AUC/time) between arms during T0–T240 (minutes).

	Suf		**C8**		**C8+Glu**		**Coc**		**Coc+Glu**		**C8+Coc**	
**(mmol/L)**	**Difference (95% CI)**	***p***	**Difference (95% CI)**	***p***	**Difference (95% CI)**	***p***	**Difference (95% CI)**	***p***	**Difference (95% CI)**	***p***	**Difference (95% CI)**	***p***
Suf			−0.27 (−0.35 • −0.19)	<0.001	−0.10 (−0.18 • −0.02)	0.02	−0.05 (−0.13 • 0.03)	0.25	0.10 (0.02 • 0.17)	0.02	−0.27 (−0.35 • −0.19)	<0.001
**C8**	0.27 (0.19 • 0.35)	<0.001			0.17 (0.09 • 0.25)	<0.001	0.22 (0.14 • 0.30)	<0.001	0.37 (0.29 • 0.44)	<0.001	0.00 (−0.08 • 0.08)	0.97
**C8+Glu**	0.10 (0.02 • 0.18)	0.02	−0.17 (−0.25 • −0.09)	<0.001			0.05 (−0.03 • 0.13)	0.20	0.19 (0.11 • 0.27)	<0.001	−0.17 (−0.25 • −0.09)	<0.001
Coc	0.05 (−0.03 • 0.13)	0.25	−0.22 (−0.30 • −0.14)	<0.001	−0.05 (−0.13 • 0.03)	0.20			0.14 (0.06 • 0.22)	0.001	−0.23 (−0.30 • −0.15)	<0.001
Coc+Glu	−0.10 (−0.17 • −0.02)	0.02	−0.37 (−0.44 • −0.29)	<0.001	−0.19 (−0.27 • −0.11)	<0.001	−0.15 (−0.22 • −0.06)	0.001			−0.37 (−0.45 • −0.29)	<0.001
**C8+Coc**	0.27 (0.19 • 0.35)	<0.001	0.00 (−0.08 • 0.08)	0.97	0.17 (0.09 • 0.25)	<0.001	0.23 (0.15 • 0.30)	<0.001	0.37 (0.28 • 0.45)	<0.001		

*Arm 1: Black was selected to represent the “control” arm*.

*Arm 2 and 4: Red and sand were arbitrarily chosen colors for C8 and Coc*.

*Arm 3 and 5: These arms correspond to 2 and 4, respectively (with glucose added). Colors are therefore the same as 2 and 4, but with lower opacity (in Figures 2, 5 they are striped, instead of changing opacity)*.

*Arm 6: Blue was chosen arbitrarily as a contrasting color*.

**Figure 4:**
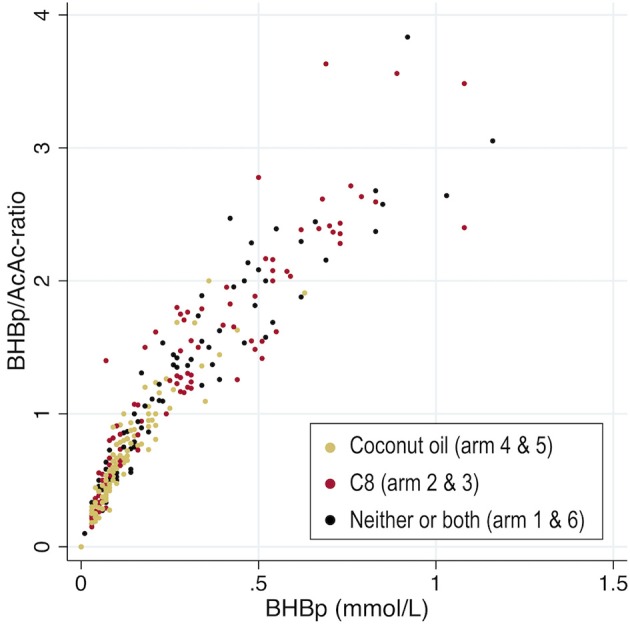
The ratio between the two plasma ketones (BHBp/AcAc), plotted against BHBp. Comparisons of arms including coconut oil vs. caprylic acid (C8) vs. neither or both. For enhanced scaling, one case in arm 2 with a ratio of 11 (0.11/0.01), is excluded from the graph. BHBp, β-hydroxybutyrate in plasma; AcAc, Acetoacetate.

To investigate potential bias from differing BHBp/AcAc-ratio in Coc vs. C8, we made a graphical comparison (Figure 4) of arms including C8 (2 & 3) vs. Coc (4 & 5) vs. neither or both (1 & 6). Our interpretation of the graph is that there is no difference between the tested oils in their paths, and this was also confirmed by comparing C8 and Coc in a linear regression with the BHBp/AcAc-ratio as the dependent variable. Although the ratio was significantly higher in C8 vs. Coc (*p* < 0.001), this difference disappeared (*p* = 0.88) when BHBp was added as a covariate, to adjust for the fact that the arms have their values distributed in different ranges of ketosis. There was a high correlation between BHBp and the BHBp/AcAc-ratio (r = 0.81, *p* < 0.0001; *n* = 360).

**Figure 5:**
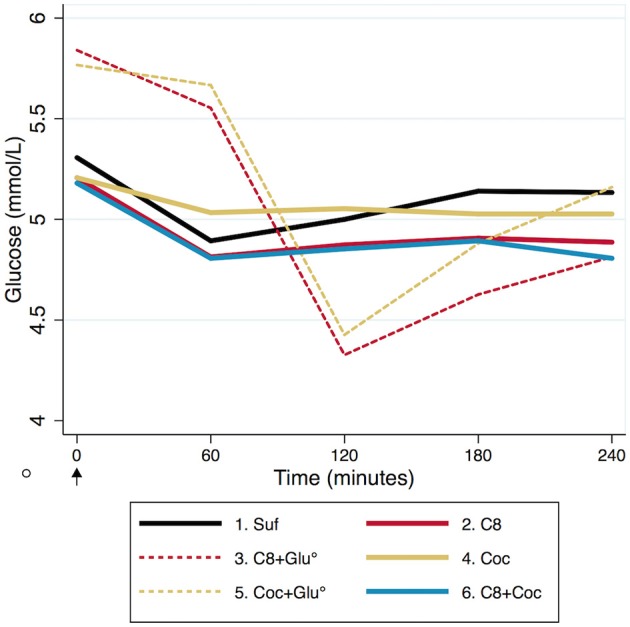
Descriptive dynamics of mean blood glucose during T0–T240 (minutes). T0 represent 12 h fasting values, except for arm 3 & 5 (striped), where glucose was ingested 15 min before the blood test performed immediately before T0, indicated by a circle. The arrow at T0 indicates the start of ingestion of coffee with fatty acids (5 mmol/L = 90 mg/dL).

**Figure 6:**
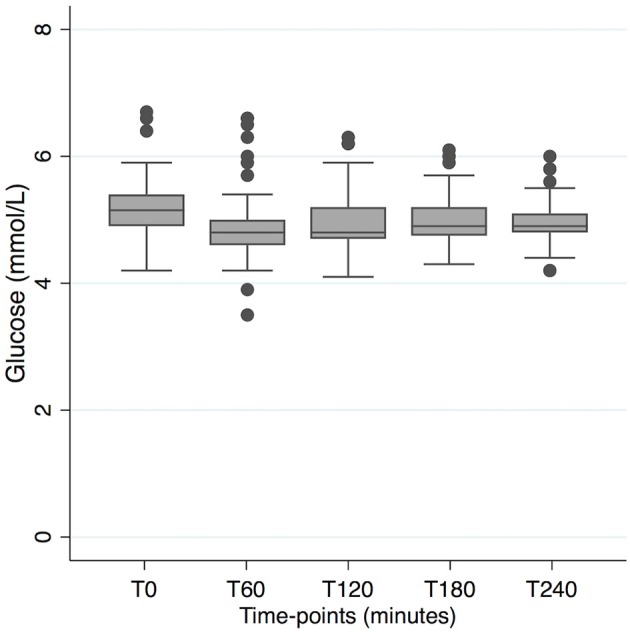
Distribution of blood glucose levels in the four arms with a 16-h non-carbohydrate window. T0 represents time for start of ingestion of fatty acids, preceded by 12 h fasting. (5 mmol/L = 90 mg/dL).

Glucose levels in plasma are described in Figures 5, 6. We compared the arms Suf and Coc (low ketosis) vs. C8 and C8+Coc (high ketosis) in a mixed regression model, controlling for repeated measures in the same individual, to investigate if glucose levels were different at any time point between the two groups. Slightly lower levels were observed in the high ketosis group at T240 (−0.17 mmol/l, 95% CI −0.33 • −0.01, *p* = 0.04), but this was not significant after Bonferroni correction. There was no difference at the other time points. Glucose was lower at all time-points T60-T240 compared to T0 in all non-glucose arms (*p* < 0.05, uncorrected). After Bonferroni correction, significance remained at all time points T60-T240 compared to T0 for C8 and C8+Coc, and at T60-T120 compared to T0 for Suf. On an individual level, the change in glucose T0–T60 had a negative association with the simultaneous change in BHBv concentrations (*p* = 0.006).

Sufficient satiety—defined as answer a/b/c in Table 4—was reported by 80–93% in the low-caloric arms at T240. Satiety was not higher in the high-caloric arms. Tolerance was in general good according to the questionnaire (Table 5). Absence of inconvenience was reported by 80–93% in the low-caloric arms, and by 60–73% in the high-caloric arms. The one report of major inconvenience, and two of the three cases of moderate inconvenience, referred to nausea. The third case of moderate inconvenience was more diffuse, and associated with tiredness. Three cases of diarrhea occurring at a later stage of a study day were reported by participants to the test leaders, and these were not captured by the questionnaire. Two of these were in the Coc arm, and one in the C8+Coc arm. Answers were missing from one participant in the C8+Coc arm.

**Table 4:** Self-reported satiety, assessed by a questionnaire directly after T240 (minutes).

**“What is the most suitable description of your hunger?”**	**Suf**	**C8**	**C8+Glu**	**Coc**	**Coc+Glu**	**C8+Coc**
a. Not hungry at all.	27%	27%	13%	33%	20%	13%
b. Modestly hungry. Lunch can wait.	13%	33%	33%	20%	27%	33%
c. It feels like the right time for lunch. Hunger appeared during the last hour.	53%	27%	33%	40%	40%	27%
d. Very hungry. Hunger appeared during the last hour.	0%	7%	13%	0%	13%	13%
e. Hungry or very hungry. Hunger has been palpable for more than one hour.	7%	7%	7%	7%	0%	7%

*Arm 1: Black was selected to represent the “control” arm*.

*Arm 2 and 4: Red and sand were arbitrarily chosen colors for C8 and Coc*.

*Arm 3 and 5: These arms correspond to 2 and 4, respectively (with glucose added). Colors are therefore the same as 2 and 4, but with lower opacity (in Figures 2, 5 they are striped, instead of changing opacity)*.

*Arm 6: Blue was chosen arbitrarily as a contrasting color*.

**Table 5:** Self-reported tolerance, assessed by a questionnaire directly after T240 (minutes).

**“Did you experience any inconvenience (e.g., nausea, upset stomach), which you attribute to the beverage you were served today?”**	**Suf**	**C8**	**C8+Glu**	**Coc**	**Coc+Glu**	**C8+Coc**
a. No	93%	87%	73%	80%	60%	67%
b. Yes, minor inconvenience.	7%	13%	27%	13%	20%	27%
c. Yes, moderate inconvenience.	0%	0%	0%	7%	13%	0%
d. Yes, major inconvenience.	0%	0%	0%	0%	7%	0%

*Arm 1: Black was selected to represent the “control” arm*.

*Arm 2 and 4: Red and sand were arbitrarily chosen colors for C8 and Coc*.

*Arm 3 and 5: These arms correspond to 2 and 4, respectively (with glucose added). Colors are therefore the same as 2 and 4, but with lower opacity (in Figures 2, 5 they are striped, instead of changing opacity)*.

*Arm 6: Blue was chosen arbitrarily as a contrasting color*.

## Discussion

We observed that ketosis was higher after intake of C8 compared to Coc or Suf. The difference between C8 and Coc was significant both with and without previous glucose intake, but glucose intake attenuated ketosis for both. This attenuation was not observed in C8+Coc (high-caloric) vs. C8 (low-caloric), indicating that it was not driven by increased energy content in general, but specifically by carbohydrates. When Coc was compared to C8+Glu or Suf, the relatively small difference in ketosis was not significant. Blood glucose was mainly stable in the non-glucose arms during the 4 h of testing. Most participants reported good tolerance and sufficient satiety.

Our results are in line with previous findings on changes in ketosis after intake of different fatty acids (11, 12), but adds information on their relative contribution in relation to fasting ketosis, and also expands the evidence to include an older sample. Extension of the non-carbohydrate window of an overnight fast—by replacing breakfast with coffee plus any of the tested fatty acids—induced a mild ketosis in the same range as the additional effect of 20 g C8 (on AUC/time). Our results suggest that consumption of MCT-supplements or coconut oil for the purpose of achieving ketosis, is optimized by combining it with time restricted feeding regarding carbohydrates. Whether a 16-h non-carbohydrate window distributed at another part of the day would give a similar response on ketosis and satiety, is a question for further studies.

The very similar BHBv paths of C8+Coc and C8 (Figure 2) suggest that the metabolization of C8 does not slow down by increased competition from 30 vs. 10 g of other fatty acids. Whether the observed difference between C8 and C8+Glu is a result of slower metabolization of C8 cannot be determined based on our study design. An alternative interpretation of the observed difference between those arms would be that anti-ketogenic effects (of the glucose intake) on pre-existing ketosis interact with the ketogenic effect of C8, i.e. the metabolization of C8 is not necessarily slower. In our data, such counteracting forces would be in the same range. Also, by comparing Coc with Coc+Glu, a similar anti-ketogenic response to the glucose intake can be observed, although not counteracted by a ketogenic effect by Coc (Figure 2). Simplified, three levels of ketosis (BHBv, AUC/time) can be identified in our data: low (0.08 mmol/L), mid-level (0.18–0.28 mmol/L), and high (0.45 mmol/L). At the low level we have Coc+Glu, characterized by including neither C8 nor a 16-h non-carbohydrate window. The observed level corresponds well with what is expected on a “normal” diet that is not carbohydrate-restricted (17), and would be interpreted as absence of nutritional ketosis. At the mid-level we find arms including either C8 or a 16-h non-carbohydrate window: Suf, C8+Glu, and Coc. In those arms BHBv was 120–240% higher compared to low. Finally, at the high level we find arms including both C8 and a 16-h non-carbohydrate window: C8 and C8+Coc. In comparison with low, BHBv here was increased by 450%. Ketosis may ideally be described as a continuous variable, but certain cut-off points may be useful in clinical or scientific settings. BHB ≥0.5 mmol/L, measured by capillary finger pricks, has been used as a cut-off for ketosis clinically (23) and as a research outcome (5, 24). As previously reported (21), exploratory analyses on our data revealed that a cut-off at 0.5 for capillary blood corresponded to 0.3 in venous blood (the outcome of the current article). Accordingly, at the mid-level some participants are above the threshold, and at the high level most of them would be defined to be in ketosis. Courchesne-Loyer et al. (25) estimated ketones to supply 8–9% of brain energy at a concentration of 0.29 mmol/L for total ketones, which roughly should correspond to the mid-level arms in our study, with C8 and C8+Coc being somewhat higher. In comparison, after adaptation to a ketogenic diet, BHB may be in the range 0.5–3 mmol/L (6).

A slight decrease in blood glucose between T0 and T60 was observed in all non-glucose arms (Figure 5). This response was not stronger for the arms where ketosis was high (C8 and C8+Coc) vs. low (Suf and Coc), suggesting that it was not solely driven by increased ketosis, although a negative association on an individual level was observed between change in BHBv and glucose during the first hour. Thereafter, blood glucose remained stable—slightly below baseline levels—illustrating an expected buffering capacity from liver glycogen, in combination with gluconeogenesis, where the glycerol back-bone from the metabolized triglycerides may have provided substrate (26). In the arms with glucose intake, an expected pattern was also observed: a rise in blood glucose, followed by an under-shoot reaching its minimum at T120, and a subsequent gradual increase back to normal levels at T240. When stable blood glucose is a target of interest, in combination with mild ketosis, coconut oil used in the absence of carbohydrates might be considered as a preferred alternative to C8-supplementation together with carbohydrate intake, as ketosis is not significantly different but blood glucose is more stable. And consequently, C8 in the absence of carbohydrates would be the optimal alternative to combine ketosis with stable blood glucose.

Almost every participant reported sufficient satiety, and interestingly satiety was not higher in the three high-caloric arms. More than half of the participants in the low-caloric arms responded “not hungry” or “lunch can wait” at the end of testing, suggesting that extended ketosis may be easily achieved. Concentrations of BHBv were on the rise—or at a plateau—at T240, which means that if a strict low-carbohydrate lunch had been served at this point, it is likely that a mild ketosis would have continued. Quantification of ketosis in such an 8-h study day could be a target for future studies. If a mild/moderate ketosis is achievable during a substantial part of the day even when dinner is composed *ad libitum*—by time restricted feeding regarding carbohydrates, and the optional supplementation with ketogenic MCT—it would increase flexibility in the implementation of ketogenic diets. In the low-caloric arms, 80–93% of the participants reported “no inconvenience,” while the remaining participants reported mild or moderate inconvenience, mainly related to gut tolerance or nausea. In a previous 90-day trial (27), 23% of the participants receiving an MCT-supplement discontinued the study due to tolerance problems, mainly gastrointestinal symptoms. A similar dropout rate was reported in a 6-months trial (19), and both these studies targeted patients with mild cognitive impairment. Daily MCT doses were 20 g and 2 × 15 g respectively, and intake was recommended to be with food. In comparison, our results do not suggest a lower level of tolerance in the administration context of our study, but whether this would be sustained in longer-term trials requires further studies. To minimize discontinuation due to tolerance problems, and to avoid a potentially stronger decrease in blood sugar than observed in our study, it may be advisable to limit the dose of C8 to 15–20 g per intake. Other oils and/or cream might be added for satiety, but should not be expected to make a substantial contribution to ketosis. Also, the metabolic responses observed may be different following long-term administration, and this is worth further investigation. Although C8 increased ketone levels, its relative contribution to ketosis would likely decrease in the context of a carbohydrate restricted ketogenic diet, where basal ketone levels are expected to be substantially higher.

Our study has some limitations, which should be considered in the interpretation and generalization of our results. The majority of the participants had normal BMI, glucose and insulin levels. A different ketogenic response might be found in a metabolically unhealthy population. Our choice of coffee as a vehicle was motivated by an intention to study the fatty acids in a real-life context, and not primarily with the goal of investigating the effects of coffee itself. Further studies should investigate possible additive or synergistic effects from caffeine. Increased levels of circulating free fatty acids could be one mechanism, by which caffeine may have boosted the ketogenic response, especially during the last hour investigated in our study, as shown by Vandenberghe et al. (18). Self-reported “sensitive gut” was an exclusion criterion, suggesting that tolerance in the general population of this age might be slightly lower than reported here. Participants were instructed to keep their usual diet, but we did not collect data on their actual food intake. However, baseline ketones (with a small number of possible exceptions) indicated that they were not on a low-carbohydrate diet. Our main outcome, BHBv, only show concentrations with one decimal, but considering its high agreement with the laboratory test (21), this shortcoming should be outweighed by benefits of the large number of time-points to capture the transient dynamics of ketosis. Another strength of our study is the complete collection of rich data, assessed under well-controlled conditions.

Previous studies (11, 12) have reported that coconut oil, C12 and C8 were associated with different ratios between the two blood ketones BHB and AcAc, raising interesting questions on potential variations in their metabolism. If such a difference is present, interpretations may be biased when only BHB is used to assess ketosis, as for the primary outcome of our study. However, our data does not support the hypothesis that the diverging ratios are caused by properties of the oils. Rather, we interpret the differing ratios as a secondary consequence of varying distribution along the range of ketosis, which in itself correlates strongly (r = 0.81, *p* < 0.0001) with the BHB/AcAc-ratio. This view is also supported by a visual interpretation of Figure 4, and consequently we do not consider our comparisons of Coc and C8 to be biased.

Differences between MCFA and LCFA have been shown at different stages of their uptake and metabolization. After intestinal uptake LCFA are incorporated into chylomicrons and transported through the lymphatic system, while MCFA can be transported to the liver via the portal vein. Ketogenesis predominantly takes place in liver mitochondria, and while MCFA can diffuse freely through the inner mitochondrial membrane, LCFA require enzymatic help from carnitine (28). The division between the alternative pathways mentioned above is best described as a proportional, rather than an absolute choice. Nevertheless, Dayrit (10) concluded after reviewing the evidence, that all MCFA in the range C6–C12 are metabolized differently from LCFA, both regarding intestinal uptake and mitochondrial entry. The specific ketogenic properties of C8—which in our data are distinct from C12, and according to previous findings (12) also from C10—thus calls for an explanation beyond the aforementioned metabolic shortcuts. Different pathways for beta-oxidation have been suggested to underlie these variations (29).

Our results should enhance the interpretation of previous and future studies on supplementation with MCT or coconut oil. We demonstrate, in line with Vandenberghe et al. (12), that it is not valid to generalize findings on C8 to coconut oil. Another important finding is that ketosis from C8 may differ substantially depending on timing and macronutritional composition of the overall food intake. The influence on brain health by MCT and coconut oil may not be limited to ketogenic pathways, as reviewed by Augustin et al. (30) and Fernando et al. (31) respectively. For the mechanistic interpretation of interventions, it therefore should be of great interest to determine to what degree participants actually achieve ketosis. Ideally this should be determined by blood testing, but when such data are not available our results can provide a rough estimation of ketosis under different conditions. For example, we show that it is unlikely that coconut oil, especially when added to a carbohydrate rich diet, would induce significant ketosis. Acute effects on cognition after intake of MCT (mainly C8) have been studied with conflicting results (32, 33). The large individual differences in magnitude and timing of the ketogenic response in our study (illustrated by Figure 3), illuminate the difficulty to detect potential acute effects. The observed opposing effect on blood glucose by BHB further complicates interpretation.

In combination with the observed signaling functions of BHB (9), the hypothesis that ketones could compensate for glucose hypometabolism in Alzheimer's disease and normal aging (3) provides a theoretical framework to motivate ketogenic interventions in the field of cognitive health. As small ketogenic diet interventions (34, 35), as well as interventions with MCT-supplements (19, 27), have shown promising results, the next step to advance the field would be to conduct larger clinical trials in the range of 3–6 months. Combining different ketogenic mechanisms like carbohydrate restriction, scheduled eating windows and MCT-supplementation, could be a strategy to enhance flexibility and adherence.

In summary, we conclude that C8-supplementation and time restricted feeding regarding carbohydrates provide two, optionally additive, strategies to achieve a mild ketosis in the context of otherwise food intake *ad libitum*. Our results on satiety and tolerance suggest that the strategies can be further investigated in longer-term ketogenic diet interventions, where they could potentially enhance compliance, by allowing a less strict carbohydrate restriction in total. In the absence of carbohydrate intake during 16 h, we observed that blood glucose remained stable in older adults. And finally, the heterogenic ketogenic properties of different MCFA highlight the importance of not using the terms MCT/MCFA without definition.

## Data Availability Statement

The datasets generated for this study are available on request to the corresponding author.

## Ethics Statement

The study involving human participants was reviewed and approved by the Regional Ethical Review Board in Stockholm, Sweden. The participants provided their written informed consent to participate in this study.

## Author Contributions

JN, SS, AS-M, UA, KN, SR, and MK conceived and designed research. KN performed experiments. JN, SS, AS-M, IK, and MD analyzed data. JN, SS, AS-M, IK, TN, and MK interpreted results of experiments. JN prepared figures and drafted manuscript. All authors edited and revised the manuscript, and approved the final version.

### Conflict of Interest

The authors declare that the research was conducted in the absence of any commercial or financial relationships that could be construed as a potential conflict of interest.

## Abbreviations

BHBv: β-hydroxybutyrate in venous whole blood
BHBp: β-hydroxybutyrate in plasma
AcAc: acetoacetate
Glu: glucose
Coc: coconut oil
C8: caprylic acid
C10: capric acid
C12: lauric acid
Suf: sunflower oil
AUC: area under the curve
MCFA: medium-chain fatty acids
MCT: medium-chain triglycerides
LCFA: long-chain fatty acids
BMI: body mass index.
